# An accurate and convenient method for *Mycoplasma pneumoniae* via one-step LAMP-CRISPR/Cas12b detection platform

**DOI:** 10.3389/fcimb.2024.1409078

**Published:** 2024-08-08

**Authors:** Tao Liu, Qing Liu, Fuqun Chen, Ying Shi, Guliya Maimaiti, Zhanhua Yang, Shutao Zheng, Xiaomei Lu, Hui Li, Zhaoyun Chen

**Affiliations:** ^1^ State Key Laboratory of Pathogenesis, Prevention, Treatment of High Incidence Diseases in Central Asian, Department of Clinical Laboratory, First Affiliated Hospital of Xinjiang Medical University, Urumqi, Xinjiang Uygur Autonomous Region, China; ^2^ State Key Laboratory of Pathogenesis, Prevention, Treatment of High Incidence Diseases in Central Asian, Clinical Medical Research Institute, The First Affiliated Hospital of Xinjiang Medical University, Urumqi, Xinjiang Uygur Autonomous Region, China

**Keywords:** *Mycoplasma pneumoniae*, LAMP, CRISPR, Cas12b, One-step, one-pot

## Abstract

**Introduction:**

*Mycoplasma pneumoniae* (MP) is the major cause of respiratory infections that threaten the health of children and adolescents worldwide. Therefore, an early, simple, and accurate detection approach for MP is critical to prevent outbreaks of MP-induced community-acquired pneumonia.

**Methods:**

Here, we explored a simple and accurate method for MP identification that combines loop-mediated isothermal amplification (LAMP) with the CRISPR/Cas12b assay in a one-pot reaction.

**Results:**

In the current study, the whole reaction was completed within 1 h at a constant temperature of 57°C. The limit of detection of this assay was 33.7 copies per reaction. The specificity of the LAMP-CRISPR/Cas12b method was 100%, without any cross-reactivity with other pathogens. Overall, 272 clinical samples were used to evaluate the clinical performance of LAMP-CRISPR/Cas12b. Compared with the gold standard results from real-time PCR, the present method provided a sensitivity of 88.11% (126/143), specificity of 100% (129/129), and consistency of 93.75% (255/272).

**Discussion:**

Taken together, our preliminary results illustrate that the LAMP-CRISPR/Cas12b method is a simple and reliable tool for MP diagnosis that can be performed in resource-limited regions.

## 1 Introduction

*Mycoplasma pneumoniae* (MP)is a small prokaryotic microorganism, which is a major cause of morbidity and mortality in children and adolescents. Notably, MP is commonly associated with community-acquired pneumonia (CAP) (Jain et al., 2015). In clinical practice, the typical symptoms of MP infection include fever, cough, sore throat, muscle pain, and others (Jiang et al., 2021). Additionally, bronchospasms, pneumonia, acute respiratory distress syndrome (ARDS), myocarditis, and meningitis have also been reported in severe cases (Narita, 2010). More than 12% of hospitalized children with MP infection are admitted to the ICU, and over 25% of patients with MP infection can develop severe pneumonia or extrapulmonary complications (Kutty et al., 2019; Rodman Berlot et al., 2023). As the manifestations of MP infection are non-specific and similar to those of other respiratory pathogens, such as the influenza virus, a misdiagnosis or delayed treatment poses a serious health threat to the public (Poddighe, 2018); therefore, it is unreliable to make a definite diagnosis of MP infection simply by clinical presentation. Moreover, the incubation period for MP is approximately 2–4 weeks (Principi and Esposito, 2001), and most patients remain unaware of the invasion of this pathogen for 14 days after infection, which greatly increases the risk of pathogen transmission in crowded places, such as schools. Therefore, MP causes frequent CAP outbreaks in school-aged children aged 5–15 years (Yiş et al., 2008). Therefore, rapid detection methods capable of accurately diagnosing MP early in the incubation period are critical for preventing outbreaks of MP-caused CAP.

Currently, the main diagnostic methods for MP infection include culture, serological tests, and polymerase chain reaction (PCR)-based methods (Waites et al., 2017). Culture-based methods provide a solid foundation for MP infection; however, culturing MP is time-consuming and relatively insensitive owing to its slow growth and fastidious nature (Leal et al., 2020). Regarding serological tests, reliable results are highly dependent on antibodies and exhibit high rates of false negatives (She et al., 2010). The results collected from PCR-based methods serve as the gold standard for MP identification with the requirement for specific equipment and skilled technicians, which are restricted to grassroots laboratories or point-of-care detection (Schmitt et al., 2013). To address this, several isothermal amplification techniques are developed for MP detection, such as loop-mediated isothermal amplification (LAMP) and recombinase polymerase amplification (RPA) (Cai et al., 2019; Fan et al., 2020). In the LAMP assay, the reaction can be performed at a constant temperature using simple operation and equipment, and the reaction time is shorter than that of PCR-based methods (Hong et al., 2023). However, much more effort is required to overcome the shortcomings of low specificity and false-positive results (Nagai et al., 2016).

Recently, clustered and regularly interspaced short palindromic repeats and their associated protein (CRISPR/Cas) systems have provided a novel method for pathogen diagnostics. Because collateral cleavage was discovered in some Cas proteins (Cas12, Cas13a, and Cas14), the CRISPR/Cas assay was modified for nucleic acid detection. Cas12 has cis-cleavage activity and cleaves targeted double-stranded DNA (dsDNA) by using a specific single CRISPR RNA (crRNA), and produces collateral trans-cleavage and non-specifically degrades single-stranded DNA (ssDNA) (Li et al., 2019), thereby the cleavage results of CRISPR/Cas12 can be visualized by using fluorophore and quencher-labeled ssDNA, indicating the presence of the target gene (Zhu et al., 2021). However, the sensitivity of the CRISPR system is low and it cannot be directly applied in clinical testing. Therefore, isothermal amplification technology was introduced to the CRISPR/Cas system, which yields a rapid, ultrasensitive, and specific detection method for MP (Deng et al., 2022; Li et al., 2022). Moreover, the CRISPR-Cas-Integrated LAMP has been applied for Pathogen-specific point-of-care (PoC) diagnostic tests (Atçeken et al., 2022). In current CRISPR-based methods, pre-amplification of the target gene and CRISPR-mediated detection are the two main steps. Therefore, the entire reaction consists of multiple manual operations that cannot avoid cross-contamination due to complicated diagnostic procedures. To address these drawbacks, a one-step, one-pot CRISPR-based nucleic acid detection platform (CRISPR-top) was developed (Li et al., 2021; Qiu et al., 2022). However, an appropriate platform for the identification of MP remains unknown.

In the present study, we integrated the LAMP assay with the CRISPR/Cas12b method to achieve a rapid, simple, sensitive, and specific detection method for MP detection (MP-LAMP-CRISPR/Cas12b). Our findings not only provide solid data for the reaction conditions, but also demonstrate the potential value of the present platform in resource-limited regions.

## 2 Materials and methods

### 2.1 Reagents and instruments

The reagents used in this study were a LAMP nuclear amplification kit (25102, TOLO Biotech, China) and dNTP mix (DN32, Hongene, China)

MgSO_4_ (B1003S, NEB, USA), Glycine (A610235, Sangon, China), SYTO-9 (S34854ThermoFisher, USA), AapCas12 (32118, TOLO Biotech, China), HOLMES ssDNA reporter (FAM) (31101, TOLO Biotech, China), Cas12b High Yield sgRNA Synthesis and Purification Kit (31904, TOLO Biotech, China), Digital PCR Mixture (MX0108, ZHENZHUN BIO, China). Nuclease-free water (R1600, Solarbio Life Sciences, China).

The instruments used in this study were as follows: Real-Time PCR system (SLAN-96S, HONGSHI, Shanghai), QuantStudio 3 real-time quantitative PCR system (QuantStudio 3, ThermoFisher, USA), QuantStudio 5 real-time quantitative PCR system (QuantStudio 5, ThermoFisher, USA), Qubit Fluorescent spectrophotometer (Qubit4, ThermoFisher, USA), and AccuMini Digital PCR system (AccuMini, ZHENZHUN BIO, China).

### 2.2 Sample preparation

The MP strains used in this study were isolated from clinically positive samples. A commercial DNA isolation kit (S20025, Sansure, Shanghai, China) was used to extract genomic DNA from MP according to the manufacturer’s instructions. To generate the recombinant plasmid pUC57-MP_PIP, the target gene was inserted into the pUC57 vector. The concentration of the recombinant plasmid was 23.5 ng/μL by using the AccuMini Digital PCR system (AccuMini, ZHENZHUN BIO, China) according to the instructions given by the manufacturers, which corresponds to a copy number of 6.54 ×10^9^ copies/μL. It was subsequently used as a standard product for the LAMP-CRISPR/Cas12b detection system.

### 2.3 LAMP Primer and sgRNA design

The Online NEB LAMP primer design tool (https://lamp.neb.com/#!/) was used to design a LAMP primer set targeting the MP gene putative intracellular protease (*PIP*, GenBank ID: CP039761.1). Cas12b sgRNA was designed based on the coding sequence of *PIP*. The primers and sgRNAs used in this study were synthesized by Sangon Biotech (Shanghai, China). Cas12b sgRNA was purified using the Cas12b High Yield sgRNA Synthesis and Purification Kit (31904, ToloBio, China) according to the manufacturer’s instructions.

### 2.4 LAMP reaction

The total volume of the LAMP reaction 25 µL, the mixture includes 2.5 µL of 10 x LAMP isothermal reaction buffer, 1 µL dNTP mix (25 mM), 2 µL MgSO_4_ (100 mM), 2.5 µL 10× MP PIP LAMP primer mix, 6 µL glycine (2M), 1 µL of Bst 2.0 DNA polymerase, 1.25 µL MP *PIP* sgRNA (10 μM), 2.5 µL template, and nuclease-free water up to 25 µL. LAMP amplification was performed at 57°C for 45 min using pUC57-MP PIP plasmid and nuclease-free DW (NTC, negative control). The products of LAMP amplification were monitored using the Applied Biosystems QuantStudio 5 Real-Time PCR System (QuantStudio 5, ThermoFisher, USA). Each reaction was repeated three times.

### 2.5 One-step LAMP-CRISPR/Cas12b reaction condition

To identify the optimum conditions for the one-step LAMP-CRISPR/Cas12b assay, the total volume of MP LAMP-CRISPR/Cas12b reaction mixture is 25 µL and prepared as follows, 2.5 µL 10×LAMP buffer, 0.8/1.0/1.2/1.4 µL dNTP mix (25 mM), 1.5/1.75/2/2.25/2.5 µL MgSO_4_ (100 mM), 2.5 µL 10 × MP *PIP* LAMP primer mix, 6 µL Glycine (2 M), 1.25 µL HOLMES ssDNA reporter (10 μM), 0.625/1.25/1.875/2.5 µL AapCas12b (10 μM), 1 µL Bst (8 U/μL), 0.625/1.25/1.875/2.5 µL MP PIP sgRNA (10 μM), 2.5 µL template and nuclease-free water up to 25 µL. The reaction was performed at 57°C for 45 min and monitored using a fluorescence reader to collect the FAM fluorescent channel signals every 30 s.

### 2.6 Ethics statement

This study was approved by the Research Ethics Committee of First Affiliated Hospital of Xinjiang Medical University (No. K202403-32). All the experiments were performed according to the relevant regulations.

### 2.7 Human nasopharyngeal swab samples

A total of 272 human nasopharyngeal swab samples were collected from The First Affiliated Hospital of Xinjiang Medical University from October 19th, 2023, to June 1st, 2024, from 140 males and 132 females (age range: 1–35 years). There were 143 MP and 129 non-MP samples (27 Influenza A virus, 12 Influenza B virus, 26 rhinovirus, 14 respiratory syncytial virus, 12 adenovirus *Adenovirus*, 2 Whooping cough, 5 Klebsiella pneumoniae, 22 Streptococcus pneumoniae, 4 Legionella pneumophila, 5 Chlamydia pneumoniae), according to clinical justification. All patients were well informed and signed the consent form. The details are shown in **Table 1**.

**Table 1:** The details of human nasopharyngeal swab samples.

Pathogen	Sampling Date	Gender		Sum
		Male	Female	
Mycoplasma pneumoniae	Oct. 2023	19	16	35
	Nov.2023	31	26	57
	May. 2024	23	24	47
	Jun. 2024	3	1	4
	Total	76	67	143
Influenza A virus	Oct. 2023	13	13	26
	Nov.2023	0	1	1
	Total	13	14	27
Influenza B virus	Oct. 2023	3	7	10
	Nov.2023	2	0	2
	Total	5	7	12
Rhinovirus	Oct. 2023	14	12	26
Respiratory syncytial virus	Oct. 2023	8	6	14
Adenovirus	Oct. 2023	3	8	11
	Nov.2023	1	0	1
	Total	4	8	12
Whooping cough	May. 2024	0	2	2
Klebsiella pneumoniae	May. 2024	3	0	3
	Jun. 2024	0	2	2
	Total	3	2	5
Streptococcus pneumoniae	May. 2024	5	7	12
	Jun. 2024	6	4	10
	Total	11	11	22
Legionella pneumophila	Jun. 2024	3	1	4
Chlamydia pneumoniae	Jun. 2024	3	2	5
Sum		140	132	272

## 3 Results

### 3.1 The workflow of MP-LAMP-CRISPR/Cas12b detection system

**Figure 1:**
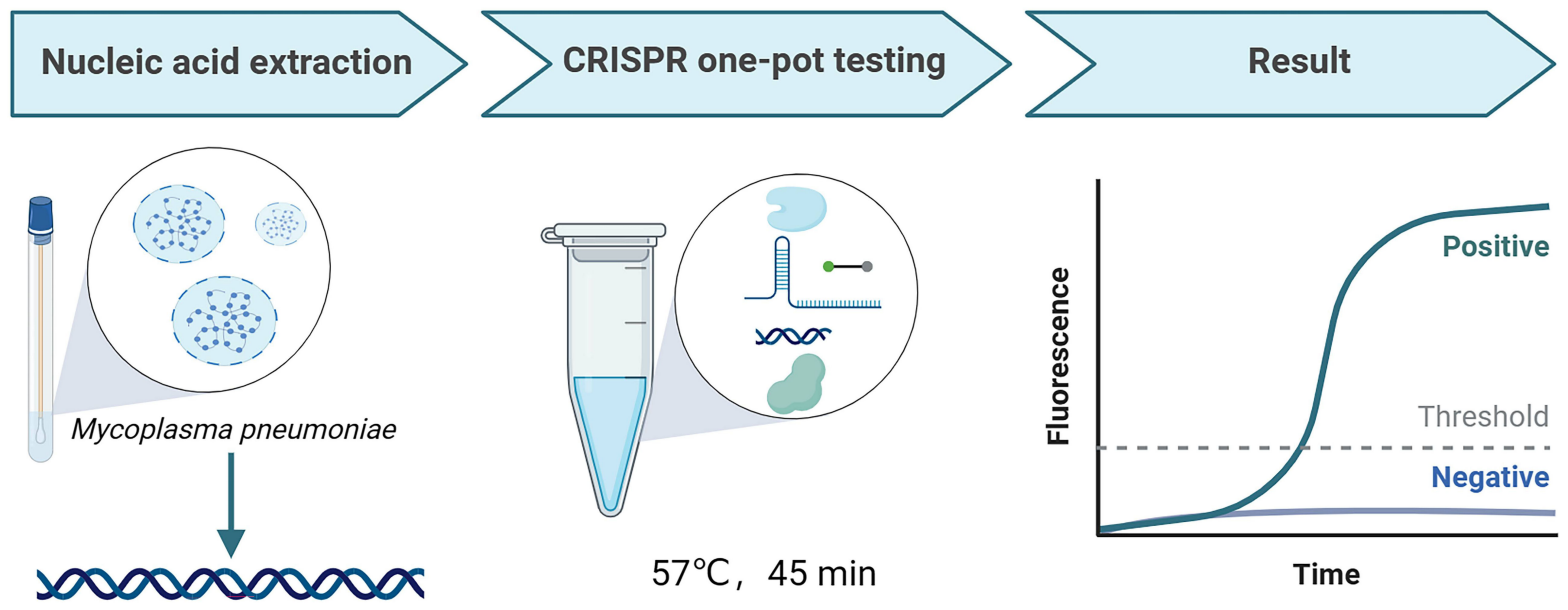
The workflow of MP-LAMP-CRISPR/Cas12b detection system. The whole assay consisted of MP nucleic acid extraction, CRISPR one-pot testing, and result readout. The MP nucleic acid amplification and detection were simultaneously performed in one-pot by one-step at a constant temperature.

The workflow of the MP-LAMP-CRISPR/Cas12b assay is shown in **Figure 1**. The MP-LAMP-CRISPR/Cas12b detection platform combines LAMP with the CRISPR/Cas12b-based detection assay, which can be performed in a single reaction step at a constant temperature for the detection of MP. The crude genomic DNA of MP was extracted using a commercial DNA isolation kit. The LAMP assay was used to amplify a target gene containing a PAM site (TTG) specific to Cas12b. Subsequently, the trans-cleavage activity of Cas12b was activated once the LAMP-amplified products were recognized by the corresponding AapCas12b/sgRNA complex, followed by non-specific cleavage of the reporter DNA that was labeled with the fluorophore 6-FAM and quencher BHQ1, resulting in the release of fluorescence, which could subsequently be detected by real-time PCR fluorescence readout or other small and portable blue light instruments. The entire test, including rapid template preparation (15 min), the LAMP-CRISPR/Cas12b reaction, and result detection (45 min), was completed within 1 h.

### 3.2 Selection and confirmation of LAMP primers

**Figure 2:**
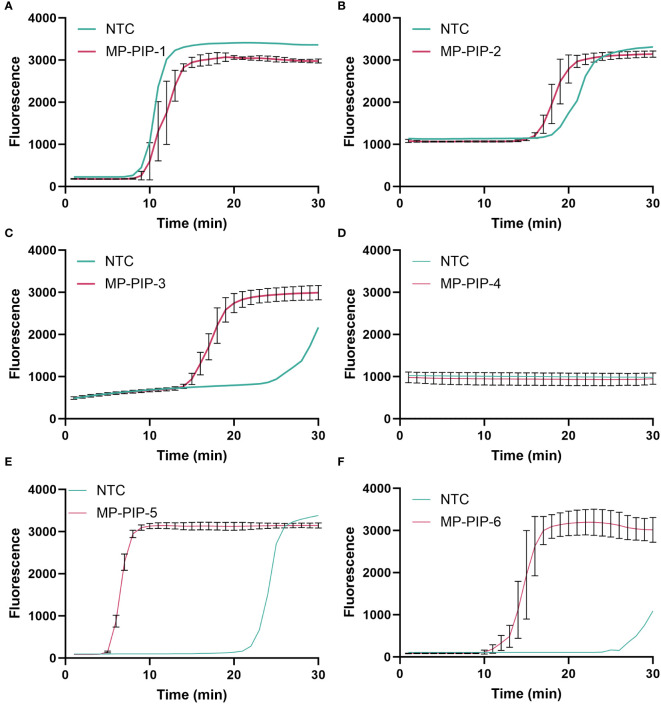
Selection of LAMP primer for *PIP* amplification. A total of Six groups LAMP primers were designed for selection. The amplification curve was presented from **(A–F)**, corresponding to MP-PIP-1 to MP-PIP-6. NTC, no template control.

putative intracellular protease (*PIP*) (GenBank: CP039761.1), which is a highly conserved gene in MP, was selected as the target gene for MP (Ratliff et al., 2014; Kanwar et al., 2018). In the present study, a plasmid containing a specific conserved sequence region of *PIP* was synthesized, and six pairs of LAMP primers (see **Table 2**) were designed by using the online NEB LAMP primer design tool (https://lamp.neb.com) based on the targeting conserved region. To explore the validity of the primers, LAMP reactions were performed at 60°C for 30 min. The LAMP primer pre-mixture was prepared by using 16 µL each of the forward inner primer (FIP) and backward inner primer (BIP), 4 µL each of the loop forward (LF) and loop backward (LB) primers, and 2 µL each of the forward outer primer (F3) and backward outer primer (B3), and 56 µL nuclease-free water. Taking the take-off time and amplification efficiency into consideration, the MP-PIP-5 primer showed higher effectiveness than the others, and without non-specific amplification product (**Figures 2A–F**). Therefore, the MP-PIP-5 primer was selected for the following reactions.

**Table 2:** The LAMP primers used in this study.

Primer	Sequences(5’-3’)	
MP-PIP-1	F3	CCATCATTATAACGCAGAAGA
	B3	GCAGCGGTATACTGGTTA
	FIP	GGTCAACAGCATACCTTGCTTTGCTAATGATCTGCACGCTACT
	BIP	TTCCGCTGAAGCCAAAGCTAAGTTAGTTTTGGACAAAGTGT
	LB	ATGTTAGAGATGGGGATTCATGTGC
MP-PIP-2	F3-2	TGCTTACAACCGCAAACA
	B3-2	GCACATGAATCCCCATCTC
	FIP-2	GCAGATCATTAGTCTTCTGCGTTACGAATCGCCAAATCACGT
	BIP-2	ACGCTACTGTACCCTGCTTTTTAATGCTAGCTTTGGCTTCAG
	LB-2	GGCGCAAAGCAAGGTATGCT
MP-PIP-3	F3-3	CCTGTTCGAATTGCCATCA
	B3-3	GCAGCGGTATACTGGTTA
	FIP-3	ACCTTGCTTTGCGCCATAAAAACGCAGAAGACTAATGATCT
	BIP-3	TGTTGACCTGATTTCCGCTGAAGGACAAAGTGTTGTCACAG
	LF-3	GCAGGGTACAGTAGCGTGC
MP-PIP-4	F3-4	ACGTCCTGTTCGAATTGC
	B3-4	AAAGCAGCGGTATACTGG
	FIP-4	GCGCCATAAAAAGCAGGGTACCATCATTATAACGCAGAAGACT
	BIP-4	CTGTTGACCTGATTTCCGCTGAGACAAAGTGTTGTCACAGC
	LB-4	ATGTTAGAGATGGGGATTCATGTGC
MP-PIP-5	F3-5	TCCAAAACTAACTTTAACCAGTA
	B3-5	CGGTGACTAATTGGTCAGG
	FIP-5	CTCCAAGTCCTTTCTTAGTTTGTCTGCTGCTTTTATTCCCCATG
	BIP-5	TAAACCCAAGGGACCAATGCGGTCAAACTCCTTCAAAACACAA
	LF-5	TTCCACCAACCTAGTAGTGTTGC
	LB-5	CTGGTTGTTTAGCAGTGGTAATGGT
MP-PIP-6	F3-6	GAAATAGACAAACTAAGAAAGGACT
	B3-6	GGTTGCTTGATGAAATTTTTTCC
	FIP-6	GCACCATTACCACTGCTAAACAAGGAGAAGTTTGTCTATAAACCCA
	BIP-6	TTGTGTTTTGAAGGAGTTTGACTTGCAAAAGTTTAACCATTTCCTTTTCG
	LB-6	ATTGCCCCTGACCAATTAGTCAC

### 3.3 Selection and confirmation of Cas12b sgRNA

**Figure 3:**
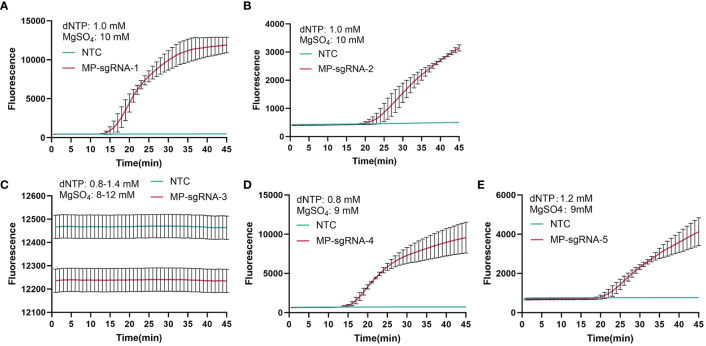
Selection of Cas12b sgRNA for *PIP* detection. A total of five Cas12b sgRNA that targeting *PIP* were designed for selection. The detection curve was presented from **(A–E)**, corresponding to MP-sgRNA-1 to MP- sgRNA-5. NTC, no template control.

Based on the amplification products of primer MP-PIP-5, five Cas12b single guide RNA (Cas12b sgRNA1-5, shown in **Table 3**) were designed and synthesized. Moreover, a LAMP-based system was used to select the sgRNA. Both the fluorescence and take-off time of sgRNA-1 were better than those of the others, and sgRNA-1 was chosen and used in the following assays (**Figures 3A–E**).

**Table 3:** The sgRNA used in this study.

sgRNA	Sequence (5’-3’)
sgRNA-1	GUCUAGAGGACAGAAUUUUUCAACGGGUGUGCCAAUGGCCACUUUCCAGGUGGCAAAGCCCGUUGAGCUUCUCAAAUCUGAGAAGUGGCAC **CACCAACCUAGUAGUGUUGC**
sgRNA-2	GUCUAGAGGACAGAAUUUUUCAACGGGUGUGCCAAUGGCCACUUUCCAGGUGGCAAAGCCCGUUGAGCUUCUCAAAUCUGAGAAGUGGCAC **UUUCCACCAACCUAGUAGUG**
sgRNA-3	GUCUAGAGGACAGAAUUUUUCAACGGGUGUGCCAAUGGCCACUUUCCAGGUGGCAAAGCCCGUUGAGCUUCUCAAAUCUGAGAAGUGGCAC **UUACCACUGCUAAACAACCA**
sgRNA-4	GUCUAGAGGACAGAAUUUUUCAACGGGUGUGCCAAUGGCCACUUUCCAGGUGGCAAAGCCCGUUGAGCUUCUCAAAUCUGAGAAGUGGCAC **GUUGUUUAGCAGUGGUAAUG**
sgRNA-5	GUCUAGAGGACAGAAUUUUUCAACGGGUGUGCCAAUGGCCACUUUCCAGGUGGCAAAGCCCGUUGAGCUUCUCAAAUCUGAGAAGUGGCAC **UUUAGCAGUGGUAAUGGUGC**

The target sequence was in bold font and underlined.

### 3.4 Optimal conditions for the MP one-step LAMP-CRISPR/Cas12b assay

#### 3.4.1 Optimal concentrations of Cas protein and sgRNA

**Figure 4:**
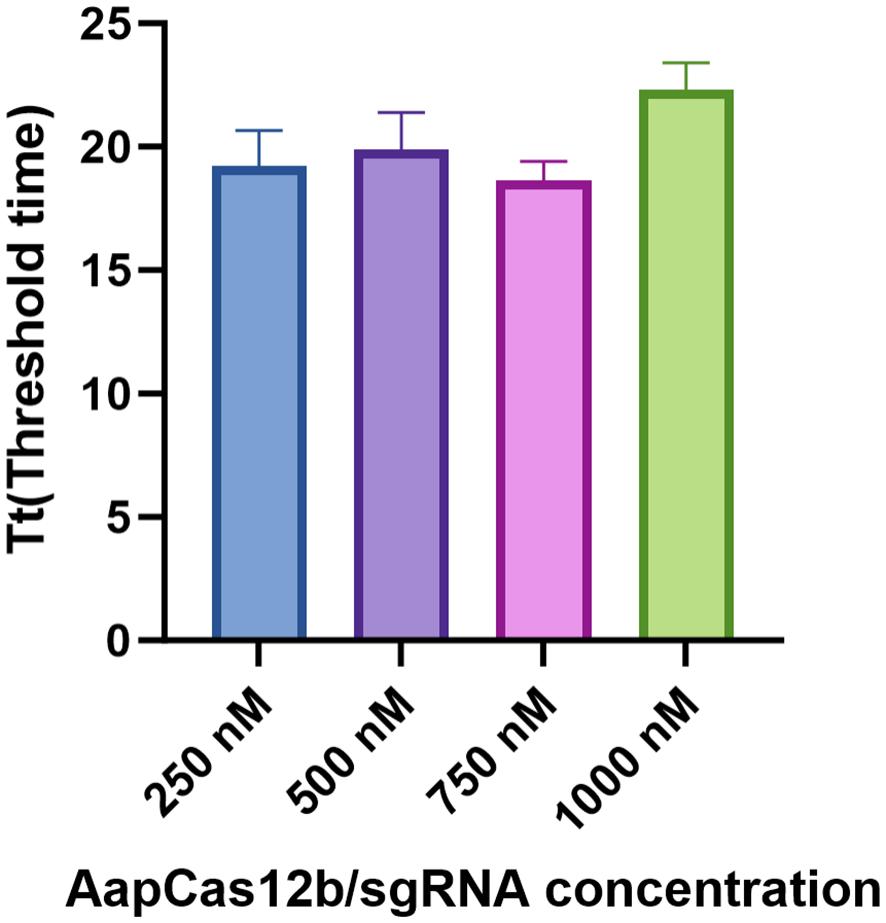
Determination of the optimal concentrations for Cas12b protein and Cas12b sgRNA. The reaction was set by using different concentrations of Cas12b and sgRNA, including 250 nM, 500 nM, 750 nM and 1000 nM respectively. The concentration was optimized according to the threshold time for the reaction. Three replications were established for each reaction.

To determine the optimal concentrations of Cas12b protein and Cas12b sgRNA, Cas12b protein and Cas12b sgRNA were diluted to four different concentrations, including 250 nM, 500 nM, 750 nM, and 1000 nM in the reaction. As shown in **Figure 2**, the threshold time at 750 nM for Cas12b protein and Cas12b sgRNA was the shortest among all concentrations (**Figure 4**). Therefore, the optimum reaction concentration of the Cas12b protein and Cas12b sgRNA was 750 nM.

#### 3.4.2 Optimal reaction temperature

**Figure 5:**
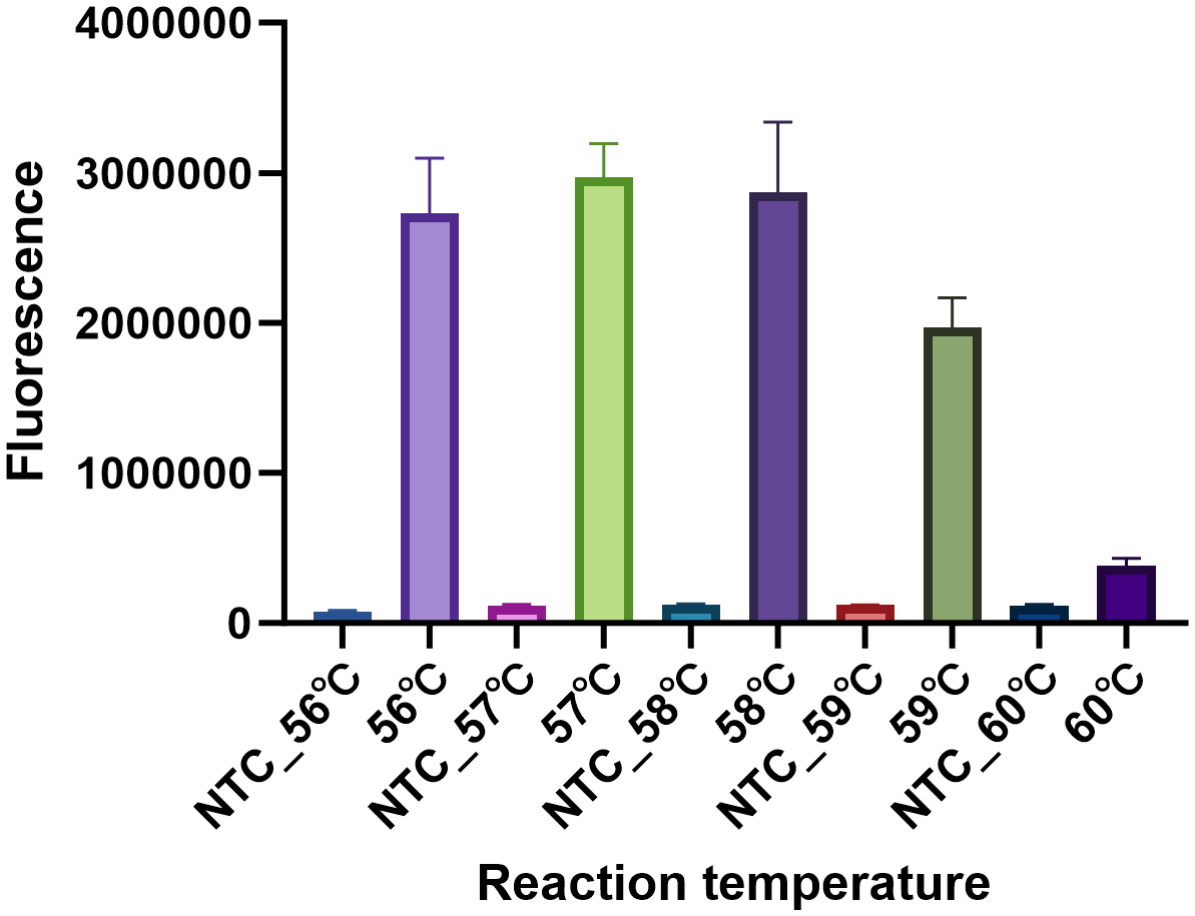
Determination of the optimal reaction temperature. To select the optimum reaction temperature, the assay was performed at 56 °C, 57 °C, 58 °C, 59 °C and 60 °C respectively. The temperature was optimized by the fluorescence intensity, three replications were established for each reaction. The fluorescence intensity of NTC at different temperatures was nearly to background.

The optimal reaction temperature was determined. The reaction temperature was set from 56°C to 60°C with 1°C increments. Nuclease-free water served as the negative control (NTC). Clearly, the reaction condition in the temperature of 57°C showed the highest fluorescence intensity than that of other temperatures (**Figure 5**). Hence, 57°C was the optimum reaction temperature of the MP CRISPR-top assay.

#### 3.4.3 Optimal ssDNA reporters

**Figure 6:**
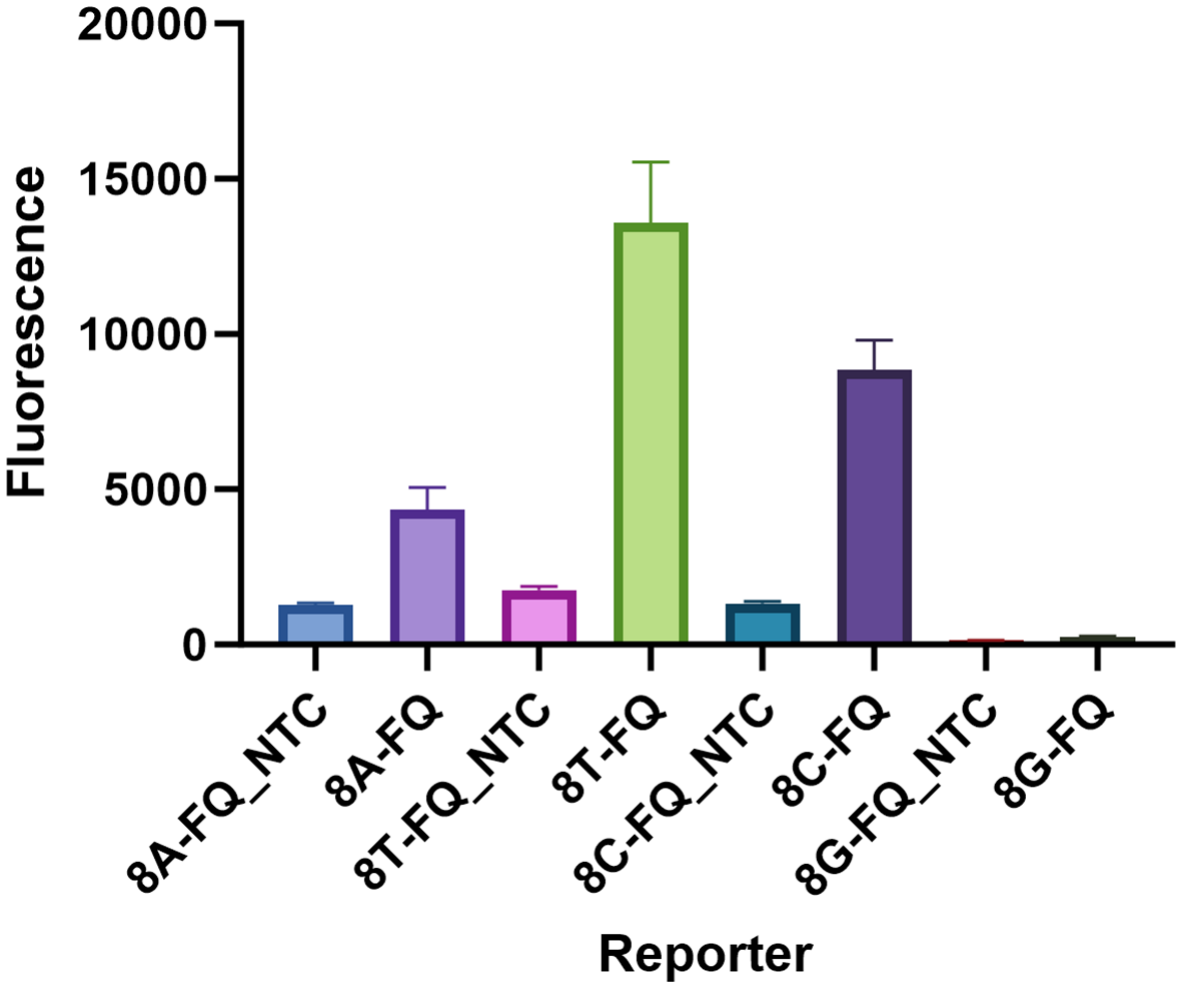
Determination of the optimal ssDNA reporter. Only 8G-FQ reporter was not worked, the left 8A-FQ, 8T-FQ, and 8C-FQ were all well-functioned. The 8T-FQ reporter was chosen based on the fluorescence intensity. Three replications were established for each reaction.

Here, a total of 4 HOLMES ssDNA-FQ reporters were used in the present examination. Based on the sequence, the reporters were named as: 8A-FQ (5’-/6-FAM/AAAAAAAA/BHQ1/-3’), 8T-FQ (5’-/6-FAM/TTTTTTTT/BHQ1/-3’), 8C-FQ (5’-/6-FAM/CCCCCCCC/BHQ1/-3’), and 8G-FQ (5’-/6-FAM/GGGGGGGG/BHQ1/-3’), respectively. Nuclease-free water served as the negative control (NTC). As shown in **Figure 6**, all the reporters were able to produce a fluorescent signal, especially 8T-FQ. Therefore, the 8T-FQ reporter was used in the SE LAMP-CRISPR-Top assay.

#### 3.4.4 Optimal chemical additives for the reaction

**Figure 7:**
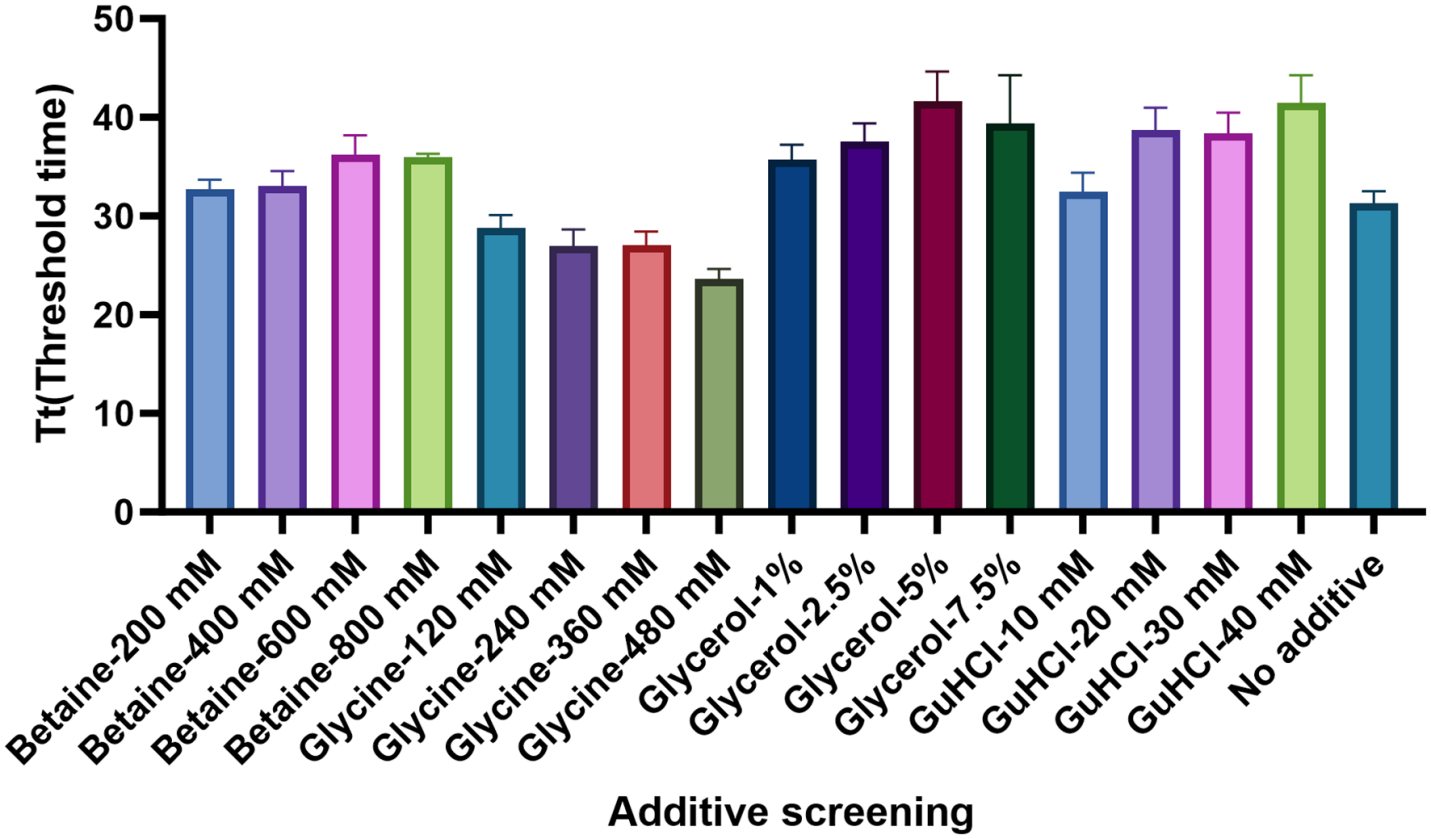
Determination of the optimal chemical additives. The optimum concentrations of Betaine, Glycine, Glycerol, and GuHCl were examined. According to the threshold time, the Glycine in the dose of 480 Mm presented the best performance. There replications were established for each reaction.

Four chemical additives at different concentrations were chosen to improve the assay performance: including betaine (200 mM, 400 mM, 600 mM, and 800 mM), glycine (120 mM, 240 mM, 360 mM, and 480 mM), glycerol (1%, 2.5%, 5%, and 7.5%), and GuHCl (10 mM, 20 mM, 30 mM, and 40 mM). As shown in **Figure 7**, the addition of 480 mM glycine significantly increased the reaction efficiency, as it produced the shortest threshold time among the additives.

### 3.5 Specificity of the MP one-step LAMP-CRISPR/Cas12b method

**Figure 8:**
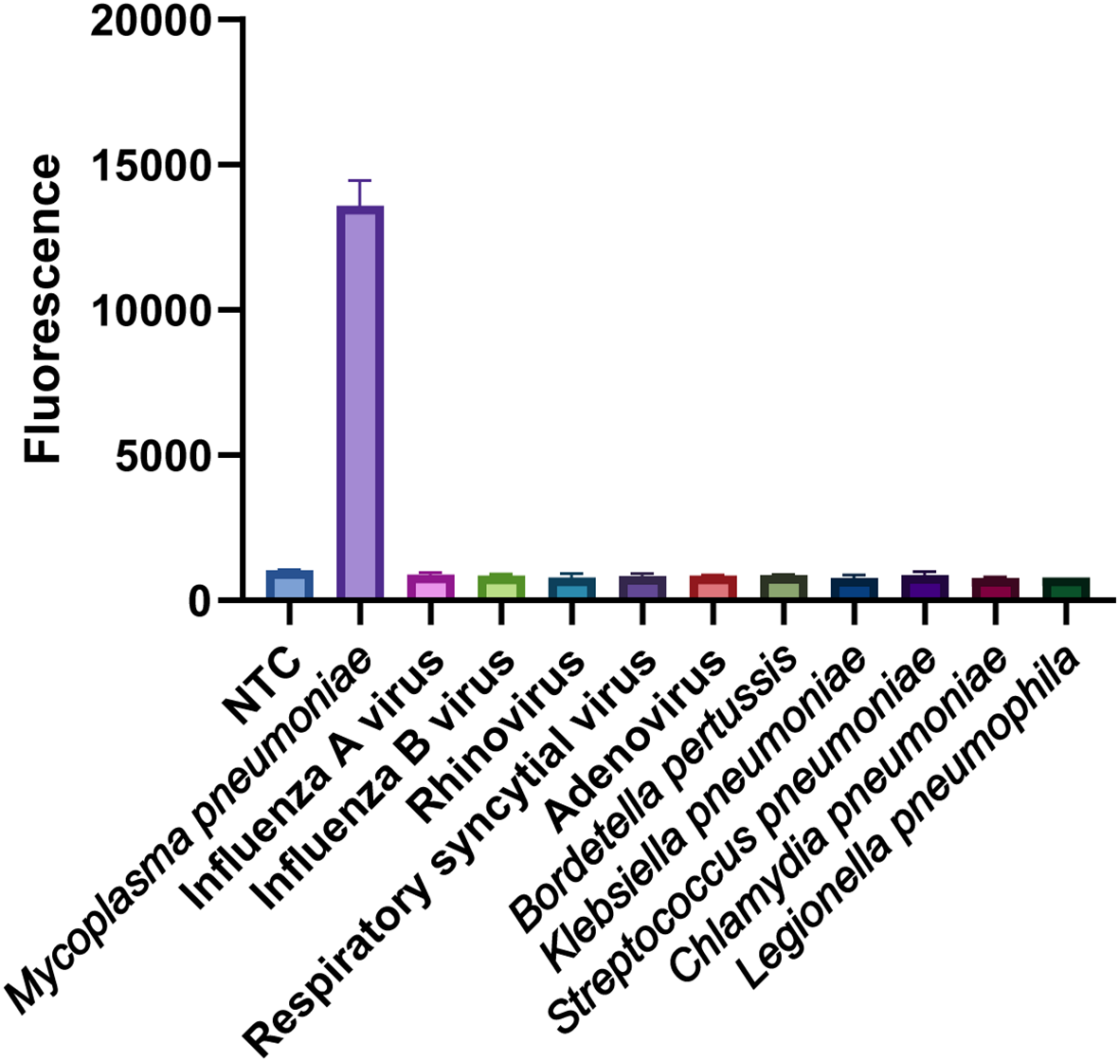
Specificity analysis of the MP one-step LAMP-CRISPR/Cas12b method. Only MP strain showed a remarkable fluorescence intensity, while the value of other non-MP strains was closely to background of NTC. Three replications were established for each reaction.

The specificity of the MP one-step LAMP-CRISPR/Cas12b assay was confirmed using various templates extracted from different pathogens, including *Mycoplasma pneumoniae*, *Influenza* A virus, *Influenza* B virus, *Rhinovirus*, respiratory syncytial virus, *Adenovirus*, Whooping cough, Klebsiella pneumoniae, Streptococcus pneumoniae, Legionella pneumophila and Chlamydia pneumoniae. All pathogens were isolated from the corresponding positive clinical samples. Nuclease-free water served as the negative control (NTC), whereas the recombinant plasmid was used as the positive control (PC). As shown in **Figure 8**, all the positive results were obtained from the PC and *Mycoplasma pneumoniae* groups, whereas all the negative results were obtained from the non-MP templates.

### 3.6 Sensitivity of the MP one-step LAMP-CRISPR/Cas12b method

**Figure 9:**
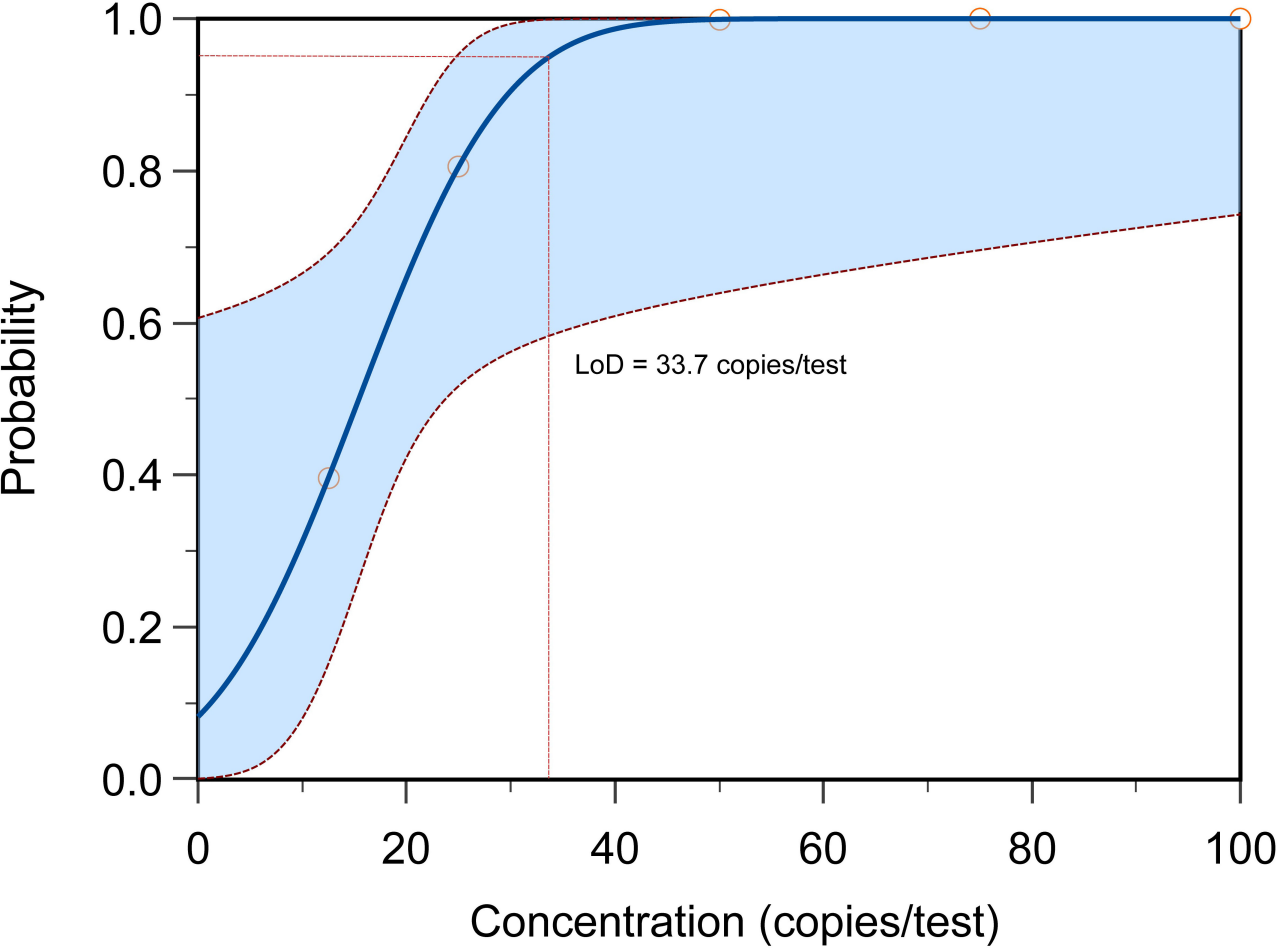
The LOD determination of MP one-step LAMP-CRISPR/Cas12b method. The LoD of present reaction achieved 33.7 copies per test with the probability about 95%, each reaction was performed in 10 replicates.

To determine the limit of detection (LOD) of the MP one-step LAMP-CRISPR/Cas12b assay, the recombinant plasmid was diluted ranging from 25 copies/μL to 100 copies/μL at intervals of 25 copies,10 replicates were necessary for per dilution of plasmid. The analytical sensitivity of the assay was 33.7 copies per reaction (**Figure 9**).

### 3.7 Validation of MP one-step LAMP-CRISPR/Cas12b method for clinical samples

A total of 272 human swab samples were tested using PCR and the MP one-step LAMP-CRISPR/Cas12b method. Taking the results of qRCR as the gold standard, the sensitivity and specificity of the MP one-step LAMP-CRISPR/Cas12b method were 88.11% (126/143), specificity of 100% (129/129), and consistency of 93.75% (255/272), the results have been presented in **Table 4**. The diagnostic results obtained using the MP one-step LAMP–CRISPR/Cas12b assay were similar to those obtained using qRT-PCR.

**Table 4:** Comparison between performance of the MP one-step LAMP-CRISPR/Cas12b method and qRCR.

MP one-step LAMP-CRISPR/Cas12b	qPCR		Sensitivity	Specificity	Consistency
	No. positive	No. negative			
Positive	126	0	88.11%	100%	93.75%
Negative	17	129			
Total	143	129			

## 4 Discussion

MP is one of the most common causative pathogens of CAP, accounting for 30–50% of CAP during the peak years. Early and rapid diagnostic methods are the key to guiding clinicians in their choice of antibiotics. However, the common methods for the identification of MP infection are culture separation, serological tests, and PCR, which are underutilized owing to shortcomings, such as time-intensiveness, insensitivity, high cost, requirement for precise instruments, and skilled technicians (Ratliff et al., 2014; Qin et al., 2023; Zhou et al., 2023). Consequently, the early, rapid, and accurate detection of MP is crucial for the diagnosis and rational selection of antibiotics.

Currently, isothermal amplification of nucleic acid technology is widely used for rapid pathogen detection of infectious diseases owing to its high efficiency and simplicity and plays an increasingly important role in promoting the on-site detection and control of pathogen diseases. LAMP was developed by Notomi et al. in 2000 (Notomi et al., 2000). In 2019, Arfaatabar et al. investigated a LAMP technique for the rapid detection of MP in clinical specimens, showing ‘substantial’ (κ=0.77) and ‘almost perfect’ (κ=0.86) congruence between the LAMP assay, culture, and PCR methods, respectively (Arfaatabar et al., 2019). However, the interpretation of the results of the LAMP assays was subjective. Thus, Wang et al. developed a LAMP coupled with a nanoparticle-based lateral flow biosensor (LFB) assay for the objective identification of MP (Wang et al., 2019a). In addition to LAMP, other isothermal amplification techniques have also been applied for the rapid detection of MP, such as multiple cross displacement amplification (MCDA) (Wang et al., 2019b), recombinase-aided amplification (RAA) (Xue et al., 2020), RPA (Jiang et al., 2022), and strand exchange amplification (SEA) (Yang et al., 2020). However, the amplified production of isothermal amplification methods often generates false-positive results; therefore, it is essential to control undesired non-specific amplification.

The specific merit of CRISPR-Cas systems is that they can be combined with isothermal amplification, which effectively solves the problem of false-positives. In 2022, Li et al. and Deng et al. developed the RPA-CRISPR/Cas12a and ERA/CRISPR–Cas12a assays for *Mycoplasma pneumoniae* detection, respectively (Deng et al., 2022; Li et al., 2022). In 2023, Jia et al. coupled the MCDA technique with a CRISPRCas12a-based biosensing system to design a novel detection platform for MP infection diagnosis and clinical application (Jia et al., 2023). Compared to Cas12a, Cas12b is ultrasensitive to mismatches between the guide RNA (gRNA) and target DNA, resulting in fewer off-target or false-positive effects and presenting a more accurate system in clinical applications (Strecker et al., 2019; Yang et al., 2023). Recently, Zhou et al. developed a CRISPR-Cas12b-based detection platform combined with RPA for MP infection that accurately distinguished MP strains from non-MP strains without cross-reactivity (Zhou et al., 2023). However, the contamination from aerosol cannot be totally avoided during the transferring of RPA products to CRISPR detection system.

The existing CRISPR-based tests for MP infection diagnosis require two separate reaction steps: (1) pre-amplification of the target nucleic acid using isothermal amplification assays and (2) detection of the resulting amplification products through CRISPR-based collateral reporter decoding. These two reaction steps require multiple manual operations and liquid handling, thus complicating the diagnostic procedures, increasing the risk of cross-contamination, and hampering their wider application in various fields.

However, application of the CRISPR system to detect MP with a one-step process has not yet been established. In this study, we established a one-step LAMP-CRISPR/Cas12b platform for MP detection. The working temperatures of Cas12b (37°C to 60°C) and LAMP (55°C to 60°C) reaction were observed to overlap (Dehghan Esmatabadi et al., 2015; Yang et al., 2016), which laid the foundation to integrate CRISPR/Cas12b with LAMP in one-pot. In the current study, the whole reaction was performed in one pot at 57°C and can be finished within 1 h. Moreover, there was no need to transfer the LAMP product to the CRISPR/Cas12b system. Therefore, our method achieved rapid detection of MP and prevented cross-contamination from multiple manual operations. As the entire assay was conducted at a constant temperature, an isothermal hot block and portable blue light equipment were sufficient for the MP-LAMP-CRISPR/Cas12b detection reaction. Moreover, the total cost of per reaction was about 10 RMB and most of the component can be pre-prepared via lyophilization. Hence, the proposed method performed efficiently in resource-limited settings. In addition, The LOD of the MP-LAMP-CRISPR/Cas12b system was 33.7 copies per reaction without any cross-reactivity with other non-MP pathogens. A preliminary evaluation using 272 clinical samples showed a sensitivity of 88.11% (126/143), specificity of 100% (129/129), and consistency of 93.75% (255/272) compared to the real-time PCR method, demonstrating that the developed method is reliable for the rapid diagnosis of MP.

## 5 Conclusion

In summary, we successfully established a rapid, simple, and accurate one-step method for MP detection by combining the LAMP assay and CRISPR/Cas12b methods in one-pot (MP-LAMP-CRISPR/Cas12b). These data indicate that the developed assay exhibits specificity and efficiency. Importantly, the MP-LAMP-CRISPR/Cas12b method has contributed to the extended detection of MP infections in various respiratory specimens, especially for point-of-care detection in resource-limited areas.

## Data availability statement

The original contributions presented in the study are included in the article/supplementary material. Further inquiries can be directed to the corresponding authors.

## Ethics statement

The studies involving humans were approved by Approval Letter of Ethics Committee of First Affiliated Hospital of Xinjiang Medical University(K202403-32). The studies were conducted in accordance with the local legislation and institutional requirements. Written informed consent for participation in this study was provided by the participants’ legal guardians/next of kin.

## Author contributions

TL: Formal analysis, Funding acquisition, Project administration, Resources, Software, Writing – original draft. QL: Conceptualization, Funding acquisition, Investigation, Methodology, Validation, Writing – original draft. FC: Methodology, Software, Writing – review & editing. YS: Data curation, Writing – review & editing. GM: Validation, Writing – original draft. ZY: Data curation, Formal analysis, Writing – review & editing. SZ: Writing – original draft. XL: Writing – review & editing. HL: Writing – review & editing. ZC: Project administration, Supervision, Visualization, Writing – review & editing.
